# Efficacy and safety of choline alphoscerate for amnestic mild cognitive impairment: a randomized double-blind placebo-controlled trial

**DOI:** 10.1186/s12877-024-05366-7

**Published:** 2024-09-19

**Authors:** Jongwook Jeon, Su Young Lee, Seunghoon Lee, Changwoo Han, Geum Duck Park, Se-Joo Kim, Jhin Goo Chang, Woo Jung Kim

**Affiliations:** 1 Department of Psychiatry, Myongji Hospital, Hanyang University College of Medicine, Goyang-Si, Gyeonggi-Do, Republic of Korea; 2 Yonsei University College of Medicine, Seoul, Republic of Korea; 3 Institute of Behavioral Science in Medicine, Yonsei University College of Medicine, Seoul, Republic of Korea; 4 Suheung Technology Research Institute, Gwacheon-Si, Gyeonggi-Do, Republic of Korea; 5 Department of Psychiatry, Severance Hospital, Yonsei University College of Medicine, Seoul, Republic of Korea; 6 Department of Psychiatry, Yongin Severance Hospital, Yonsei University College of Medicine, Yongin-Si, Gyeonggi-Do, Republic of Korea

**Keywords:** Mild cognitive impairment, Choline alphoscerate, SHCog™, Cognitive function, Memory, Language

## Abstract

**Background:**

Effective interventions for overall healthy subjects with mild cognitive impairment are currently limited. Choline alphoscerate (alpha glyceryl phosphorylcholine, αGPC) is a choline-containing phospholipid used to treat cognitive function impairments in specific neurological conditions. This study aimed to investigate the efficacy and safety of αGPC in individuals diagnosed with mild cognitive impairment.

**Methods:**

In this multicenter, randomized, placebo-controlled trial, 100 study subjects with mild cognitive impairment underwent a double-blind SHCog™ soft capsule (600 mg αGPC) or placebo treatment for 12 weeks. The primary efficacy outcome included changes from baseline on the Alzheimer’s Disease Assessment Scale–cognitive subscale (ADAS-cog). Safety assessments included regular monitoring of adverse events, and clinical laboratory tests were conducted at baseline and the end of the trial.

**Results:**

After 12 weeks of αGPC treatment, the ADAS-cog score decreased by 2.34 points, which was significantly greater than the change observed in the placebo group. No serious AEs were reported, and no study subjects discontinued the intervention because of AEs. There was no significant difference in incidence rate of AEs between the αGPC group and the placebo group.

**Conclusion:**

This study suggests that αGPC is a safe and effective intervention for improving cognitive function in study subjects with mild cognitive impairment.

**Trial registration:**

Clinical Research Information Service; Osong (Chungcheongbuk-do): Korea Centers for Disease Control and Prevention, Ministry of Health and Welfare (Republic of Korea); KCT0008797; A 12-week, multicenter, randomized, double-blind, placebo-controlled human application study to evaluate the efficacy and safety of SH_CAPK08 on cognitive function improvement in mild cognitive decline.

**Supplementary Information:**

The online version contains supplementary material available at 10.1186/s12877-024-05366-7.

## Background

Dementia is a major global health challenge that imposes substantial societal burdens and costs. According to a report by the World Health Organization in 2021 [1], the number of people living with dementia was estimated to be approximately 50 million and is anticipated to triple by 2050. Mild cognitive impairment (MCI) is considered a transitional state between normal cognitive aging and dementia [2, 3]. MCI is characterized by a noticeable cognitive decline with limited disruption of instrumental activities of daily living but does not meet the criteria for dementia [3]. In particular, amnestic MCI (aMCI), which features prominent memory impairment, has a higher rate of progression to Alzheimer’s disease (AD) dementia [4]. The progression rate from MCI to dementia over a short time is 20–40%, with longer-term progression spanning 60–100% over 5–10 years [4, 5]. Identifying effective interventions for MCI is important to delay or prevent dementia and improve the quality of life of affected individuals.

Numerous pharmacological choices, such as cholinesterase inhibitors and dietary supplements, along with non-pharmacological interventions, such as exercise and cognitive training, have been proposed as potential treatments for MCI [6–8]. However, clinical trials have yielded inconsistent outcomes, often showing minimal or negligible effects. With respect to cholinesterase inhibitors, the treatment of choice for AD dementia, a previous study demonstrated cognitive improvement of approximately a 1.0-point decrease in the Alzheimer’s Disease Assessment Scale–cognitive subscale (ADAS-cog) score in patients with MCI. However, adverse effects (AEs) in the treatment group were more than double that in the placebo group, and compliance was low (55% of participants who received donepezil) [9]. In a large-scale randomized control trial (RCT), where the primary outcome measure was the progression rate to AD, no significant difference was observed between the donepezil group and the placebo group, although AEs were significantly more prevalent in the donepezil group [10]. Because of these reason, regular exercise is the only recommended management option for patients with MCI according to the practical guidelines of the American Academy of Neurology [6].

Choline alphoscerate (alpha-glyceryl-phosphorylcholine, αGPC) is a choline-containing phospholipid that is often used as a dietary supplement. Choline passes through the blood–brain barrier, which results in increased choline levels. Choline is a precursor of acetylcholine, an essential neurotransmitter for memory and learning. In animal experiments, choline played a pivotal role in brain development, particularly in hippocampal maturation [11]. Previous clinical studies have shown that αGPC is effective in managing cognitive decline of vascular origin and in AD dementia [12–15]. Further, clinical evidence suggests that choline-enriched multi-nutrient dietary intervention may have a beneficial impact in managing MCI due to AD [16]. A recent review concluded that αGPC, either alone or in combination with the ChE-I donepezil, can improve cognition, functional, and behavioral status in patients with AD and other neurological dementia disorders [17]. These studies suggest that αGPC may be an effective management intervention for MCI.

Therefore, it is important to conduct clinical trials to examine the effects of αGPC on MCI and determine its potential as a viable treatment option. To this end, we conducted a 12-week multicenter, randomized, double-blind, placebo-controlled trial.

## Methods

### Settings

This 12-week, multicenter, randomized, double-blind, placebo-controlled, parallel-group trial was conducted at two hospital sites in the Republic of Korea. The study protocol (versionV1.0, June 24th, 2021) complied with the Declaration of Helsinki and was approved by the Institutional Review Board of the conducting institutions (Yongin Severance Hospital; 9–2021-0085, Myongji Hospital; 2021–07-035). The study is registered in the Clinical Research Information Service. Osong (Chungcheongbuk-do): Korea Centers for Disease Control and Prevention, Ministry of Health and Welfare (Republic of Korea); 2010; 14/09/2023; KCT0008797; A 12-week, multicenter, randomized, double-blind, placebo-controlled human application study to evaluate the efficacy and safety of SHCog™ on cognitive function improvement in mild cognitive decline.

### Study Subject

One hundred study subjects were recruited through advertisements. Overall healthy subjects who experienced cognitive decline were tested for aMCI by a certified clinician using neurocognitive tests. Exclusion of dementia were carried out through evaluation by psychiatrists. The inclusion criteria were participants aged between 55 and 85 years old and a decline of 1.0 standard deviation or more in either memory scores (Word List Memory of Word List Recall or Word List Recognition) on the Korean version of the neuropsychological assessment developed by the Consortium to Establish a Registry for Alzheimer’s Disease, or on the Seoul Verbal Learning Test (SVLT) of the Seoul Neuropsychological Screening Battery.

The exclusion criteria were as follows: 1) patients with concurrent neurodegenerative conditions, such as AD or Parkinson's disease; 2) current mental disorders (e.g., major depressive disorder, schizophrenia, alcohol use disorder)according to DSM-5 criteria; 3) patients who had taken medications known to affect cognitive function within 4 weeks before the initial visit (all types of psychotropics including antidepressants, nootropics, supplements for brain function) 4) ongoing treatment for severe immune, respiratory, gastrointestinal/hepatic/biliary, renal/urinary, neurological, musculoskeletal, and infectious diseases or malignancies; 5) a history of head trauma with loss of consciousness within 6 months before the initial visit; 6) a history of cardiovascular disease within 6 months before the initial visit; 7) vitamin E supplementation exceeding 400 IU per day or anticipated inability to reduce the dosage; 8) use of estrogen replacement therapy (excluding local applications) within 2 months before the initial visit; 9) use of dietary supplement related to cognitive improvement within 2 months before the initial visit; 10) thyroid disease with thyroid-stimulating hormone (TSH) levels below 0.1 μIU/mL or above 10 μIU/mL; 11) creatinine levels exceeding twice the normal upper limit; 12) aspartate transaminase (AST) or alanine aminotransferase (ALT) levels exceeding three times the normal upper limit; 13) uncontrolled hypertension and diabetes; 14) sensitivity or allergy to the ingredients of the investigational product; 15) pregnancy, breastfeeding, or planning to get pregnant during the study period; 16) participation in any other interventional clinical trial (including clinical trials other than the current study) within 3 months before the initial visit, or planned participation in any other clinical trial (including clinical trials other than the current study) after the start of the current study. All enrolled subjects were provided written informed consent after receiving a complete description of the study protocol.

### Randomization and blinding

A block randomization method was used for randomization. To ensure balanced randomization between the αGPC group and the placebo group, the ratio of participants in each group was set to 1:1. The randomization table was generated using the SAS software randomization program, which applied a permutation of random numbers to the sequential test subject numbers, starting from number one. Drug labeling was performed according to the randomization table, and the labeled drugs were supplied to the institutions before the start of the trial.

To maintain blinding, the assignment details (information about blinding) for each group were securely managed by the trial coordinator. Except in cases where it was necessary because of significant adverse drug reactions, the blinding codes were not disclosed until the end of the trial. The investigators administered the randomly assigned drug corresponding to the allocated blinding code to the eligible participants during the treatment phase human application phase of the trial. To maintain blinding, reserves (specific to each unique code) were used in cases of shortage or damage to the drugs. Throughout the study, there were no occurrences of unblinding during the trial period.

### Raw material

SHCog™ is a branded α-GPC, prepared from Soybean lecithin and is highly viscose. Materials used for the manufacturing process and residual solvent standards comply with food regulations of Korea. The raw materials were stored at room temperature until test product is prepared as a soft capsule format.

### Interventions

Enrolled subjects were administered αGPC or placebo once daily with adequate water for a duration of 12 weeks. Based on the choline dosage recommended by the National Academies Press, and considering previous studies, a single dose of the drug was determined to contain SHCog™ (600 mg of αGPC). Enrolled subjects were allowed to continue taking medications and dietary supplements that were deemed unlikely to affect the interpretation of the results. However, the following medications were prohibited during the trial because of their potential to influence the interpretation of the results: 1) medications that affect cognitive function (e.g., antipsychotics, tricyclic antidepressant, neurodegenerative disease drugs, nootropics), 2) vitamin E supplementation exceeding 400 IU per day, 3) estrogen replacement therapy (excluding local applications), and 4) dietary supplements associated with cognitive improvement.

### Outcome assessments

The primary outcome was a change in the total score Alzheimer’s Disease Assessment Scale–cognitive subscale (ADAS-cog score). The Alzheimer Disease Assessment Scale (ADAS) consists of cognitive and noncognitive sections. ADAS-cog, which is cognitive section of ADAS, aims to assess various cognitive domains in dementia patients, including memory, language abilities, and executive function, among others [18, 19]. We additionally assess concentration abilities through an item in noncognitive section. The secondary outcome assessment included the Korean version of the Montreal Cognitive Assessment (MoCA-K) [20], the Visual Continuous Performance Test (Visual C.P.T) [21], the Korean-Color Word Stroop Test (K-CWST) [22], Seoul-Instrumental Activities of Daily Living (S-IADL) [23], Subjective Cognitive Decline Questionnaire (SCD-Q) [24], and the Korean version of the Short Form Geriatric Depression Scale (SGDS-K) [25]. These outcomes were assessed at baseline and 12 weeks after baseline.

### Safety assessment

Safety assessments included monitoring of AEs, clinical laboratory tests (hematological/blood chemistry and urinalysis), vital signs (blood pressure and pulse rate), and physical measurements (body weight). These assessments were conducted at screening (2 weeks before baseline), baseline, 6 weeks after baseline, and 12 weeks after baseline.

### Sample size and power analysis

A sample size of 35 per treatment group was estimated to assess the effectiveness of αGPC, compared with that of placebo, using primary efficacy variables (ADAS-cog score), with 80% power at a significance level of 0.05. To account for a potential dropout rate of 30%, we enrolled 50 participants in each group, resulting in a total enrollment of 100 individuals across both treatment groups.

### Statistical analysis

Outcome analysis was carried out based on an intention-to-treat (ITT) dataset, defined as the subset of participants who received at least one dose of medication after randomization. The statistical analysis was conducted using SAS® software (Version 9.4, SAS Institute, Cary, North Carolina, USA).

To evaluate efficacy variables, including the ADAS-cog score, MoCA-K, visual C.P.T, K-CWST, S-IADL, SCD-Q, and SGDS-K, intra-group comparisons of pre- and post-intake changes were performed using paired t-test. The difference in extent of changes between the αGPC group and the placebo group at each time point was assessed using two sample t-test or Wilcoxon rank-sum test. Demographics and results of biological tests (Laboratory examination, measuring vital signs and body weight) were analyzed using a two-sample t-test or the Wilcoxon rank-sum test. Chi-square or Fisher's exact test was used to analyze categorical data.

## Results

### Baseline characteristics

**Fig. 1:**
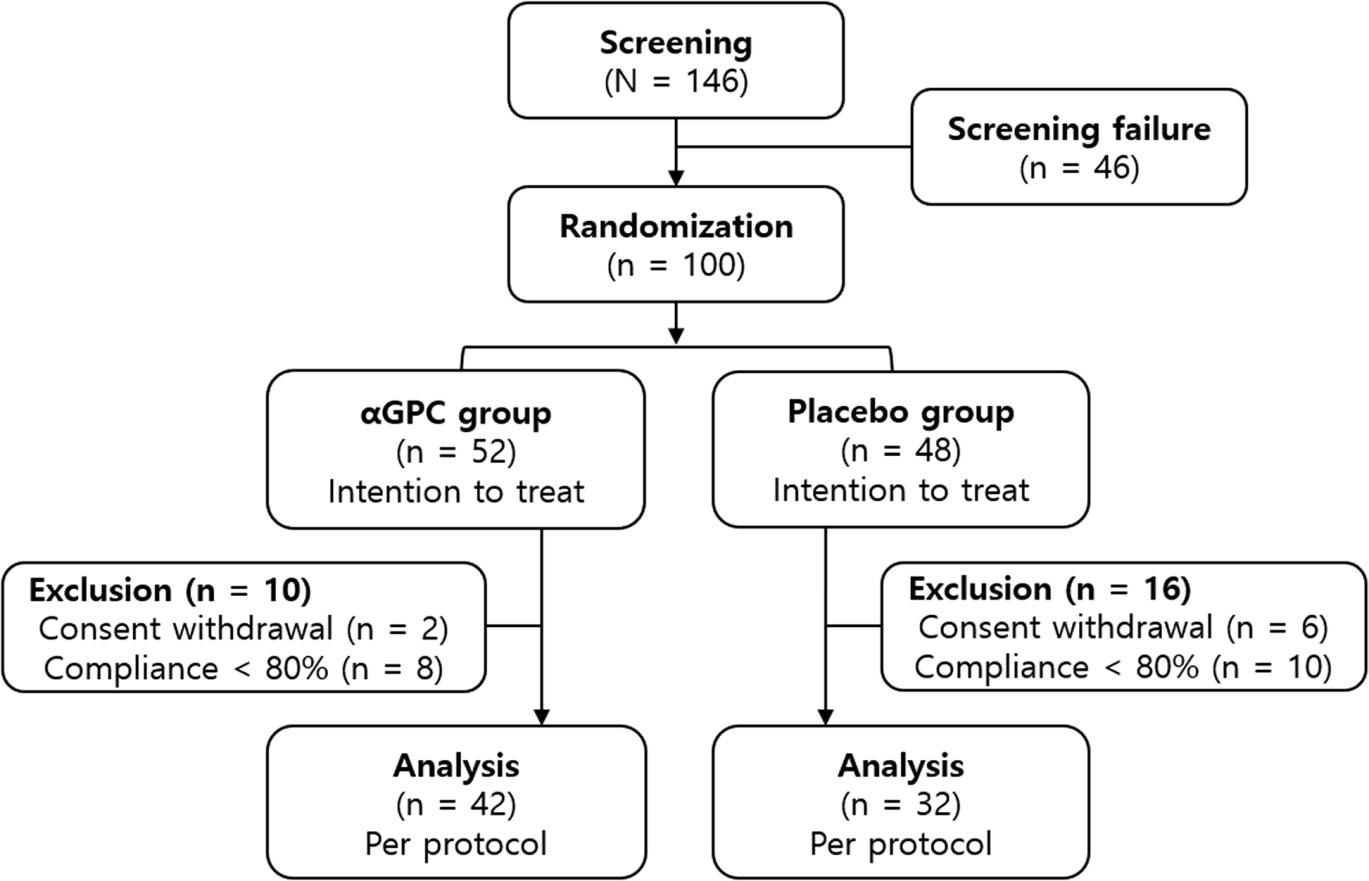
The CONSORT flowchart. **αGPC**, Choline alphoscerate

One hundred subjects were enrolled in this study, of whom 52 received αGPC and 48 received placebo (Fig. 1). All 100 subjects were included in the ITT population, whereas 74 subjects (74%) were included in the per-protocol population. For the primary and secondary outcomes, 42 subjects in the αGPC group and 32 subjects in the placebo group were analyzed.

Table 1 summarizes baseline characteristics of study participants. The αGPC and placebo groups showed similar distributions in terms of sex, age, education level, smoking and alcohol status, exercise habits, and stress severity. The results of all neuropsychological tests are presented in Table 2.

**Table 1:** Baseline demographic and clinical characteristics of the participants

	**αGPC (*N* = 42)**	**Placebo (*N* = 32)**	*P*-value
	Mean (*SD*) or N (%)	Mean (*SD*) or N (%)	
Sex			
Male	12 (28.57)	8 (25.00)	0.732
Female	30 (71.43)	24 (75.00)	
Age (years)	70.52 (9.05)	71.75 (8.43)	
50–59	7 (16.67)	4 (12.50)	0.555
60–69	11 (26.19)	8 (25.00)	
70–79	16 (38.10)	15 (46.88)	
≥ 80	8 (19.05)	5 (15.63)	
Education level			
No education	3 (7.14)	3 (9.38)	0.866
Elementary school graduate	10 (23.81)	6 (18.75)	
Middle school graduate	6 (14.29)	3 (9.38)	
High school graduate	18 (42.86)	13 (40.63)	
College or University graduate	4 (9.52)	5 (15.63)	
University postgraduate	1 (2.38)	2 (6.25)	
Smoking status			
Never smoker	34 (80.95)	28 (87.50)	0.513
Quit (≥ 1 year)	6 (14.29)	2 (6.25)	
Quit (< 1 year)	1 (2.38)	0 (0.00)	
Current smoker	1 (2.38)	2 (6.25)	
Alcohol intake			
No	34 (80.95)	25 (78.13)	0.764
Yes	8 (19.05)	7 (21.88)	
Exercise			
No	10 (23.81)	1 (3.13)	0.118
1–2 times/week	6 (14.29)	8 (25.00)	
3–4 times/week	10 (23.81)	9 (28.13)	
5–6 times/week	9 (21.43)	10 (31.25)	
Daily	7 (16.67)	4 (12.50)	
Stress severity			
None	4 (9.52)	6 (18.75)	0.501
Mild	29 (69.05)	20 (62.50)	
Moderate	8 (19.05)	4 (12.50)	
Severe	1 (2.38)	2 (6.25)	

*αGPC* Choline alphoscerate

**Table 2:** Major results of neuropsychological tests before and after 12 weeks of choline alphoscerate (αGPC) and placebo administration

	αGPC group (*N* = 42)			Placebo group (*N* = 32)			*P*-value
	Mean (*SD*)			Mean (*SD*)			
	Baseline	Week 12	Change	Baseline	Week 12	Change	
ADAS-cog							
Total score	10.40 (4.33)	8.06 (4.28)	-2.34 (3.26)	9.14 (3.33)	8.17 (3.00)	-0.97 (2.32)	0.048^‡^
Memory domain	7.79 (3.43)	6.04 (3.64)	-1.75 (2.63)	6.67 (2.20)	6.01 (2.25)	-0.66 (1.67)	0.034^‡^
Total score + concentration domain of ADAS-noncog	10.45 (4.30)	8.06 (4.28)	-2.39 (3.25)	9.20 (3.39)	8.26 (2.98)	-0.94 (2.37)	0.037^‡^
MoCA-K							
Total score	21.24 (3.74)	22.95 (4.23)	1.71 (2.65)	21.88 (3.75)	22.25 (4.39)	0.38 (2.61)	0.105^§^
Visuospatial/Executive	3.98 (1.14)	4.21 (0.92)	0.24 (1.03)	4.00 (1.05)	4.09 (0.96)	0.09 (0.89)	0.716^§^
Attention	4.71 (1.35)	5.00 (1.15)	0.29 (1.27)	5.25 (0.92)	4.94 (1.22)	-0.31 (1.35)	0.074^§^
Language	2.33 (0.82)	2.45 (0.59)	0.12 (0.74)	2.19 (0.82)	2.31 (0.78)	0.13 (0.77)	0.882^§^
Abstraction	1.00 (0.77)	1.10 (0.82)	0.10 (0.62)	0.97 (0.82)	0.81 (0.78)	-0.16 (0.77)	0.102^§^
Delayed recall	0.86 (0.95)	1.64 (1.65)	0.79 (1.41)	1.00 (1.41)	1.81 (1.73)	0.81 (1.42)	0.844^§^
Orientation	5.74 (0.54)	5.71 (0.83)	-0.02 (0.64)	5.75 (0.62)	5.66 (0.83)	-0.09 (0.59)	0.585^§^
Naming	2.38 (0.94)	2.60 (0.77)	0.21 (0.42)	2.56 (0.76)	2.47 (0.80)	-0.09 (0.39)	0.003^§^
K-CWST							
Time of word naming (s)	15.35 (3.72)	14.16 (2.79)	-1.19 (3.10)	15.21 (5.42)	13.55 (3.20)	-1.66 (3.58)	0.548^§^
Time of color naming (s)	27.64 (12.22)	26.19 (11.99)	-1.45 (9.18)	24.57 (12.80)	24.30 (9.49)	-0.27 (6.19)	0.048^§^
Time of congruent word naming (s)	15.33 (3.76)	14.93 (4.23)	-0.41 (3.34)	14.96 (5.09)	14.20 (4.42)	-0.76 (3.64)	0.832^§^
Time of incongruent word naming (s)	20.72 (12.43)	20.17 (13.09)	-0.55 (7.16)	17.43 (8.75)	17.40 (7.89)	-0.03 (5.77)	0.635^§^
Time of incongruent color naming (s)	72.26 (45.20)	65.90 (47.57)	-6.36 (24.36)	60.09 (31.07)	56.37 (30.15)	-3.73 (13.47)	0.604^§^
Error of word naming (n)	0.60 (3.70)	0.00 (0.00)	-0.60 (3.70)	0.00 (0.00)	0.03 (0.18)	0.03 (0.18)	0.100^§^
Error of color naming (n)	0.86 (1.84)	0.67 (1.96)	-0.19 (1.52)	0.59 (1.27)	0.47 (0.88)	-0.13 (0.87)	0.879^§^
Error of congruent word naming (n)	0.05 (0.22)	0.07 (0.34)	0.02 (0.41)	0.16 (0.72)	0.06 (0.25)	-0.09 (0.78)	0.976^§^
Error of incongruent word naming (n)	0.19 (0.59)	0.19 (0.63)	0.00 (0.66)	0.16 (0.57)	0.19 (0.47)	0.03 (0.47)	0.603^§^
Error of incongruent color naming (n)	2.29 (3.13)	1.79 (2.68)	-0.50 (1.67)	1.03 (1.64)	0.59 (1.04)	-0.44 (1.01)	0.678^§^

Changes between the baseline and week 12 scores were evaluated within each group using the paired t-test. Change values are highlighted in bold for outcomes that were statistically significant (*p* < 0.05). Differences in change values between the αGPC and placebo groups were assessed using either the Wilcoxon rank-sum test or the two-sample t-test, and the respective *p*-values are provided. Statistically significant *p*-values are shown in bold. ‡:Two sample t-test; §: Wilcoxon rank sum test

*αGPC* Choline alphoscerate, *ADAS-cog* Alzheimer's Disease Assessment Scale-Cognitive Subscale, MoCA*-K* Korean version of the Montreal Cognitive Assessment, *K-CWST* Korean-Color Word Stroop Test

### Primary outcome (ADAS-cog score)

**Fig. 2:**
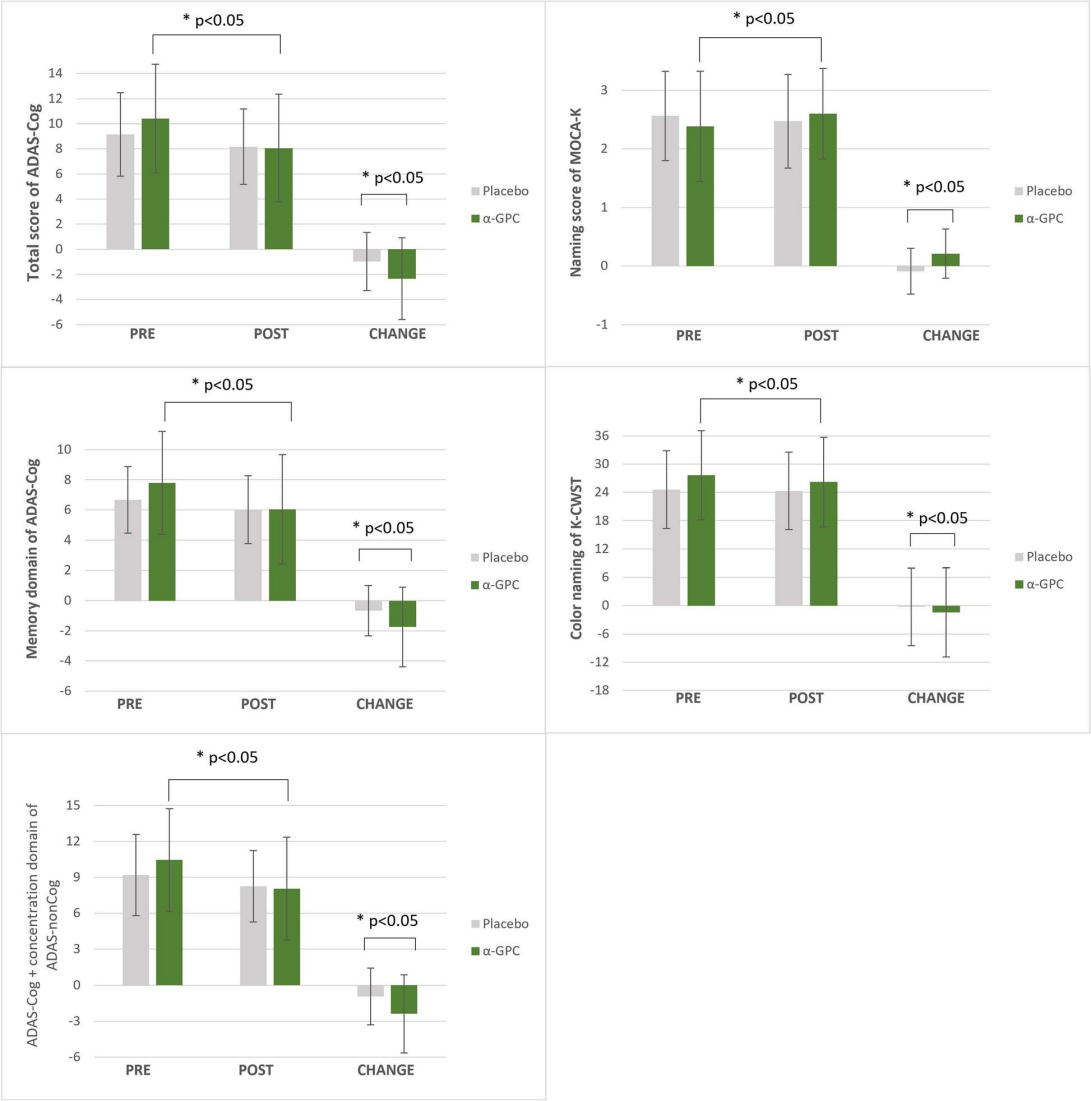
Results of the tests showing significant differences between the αGPC group and placebo group. ***αGPC***, Choline alphoscerate ***ADAS-cog,*** Alzheimer's Disease Assessment Scale-Cognitive Subscale; **MoCA*-K,*** Korean version of the Montreal Cognitive Assessment; ***K-CWST,*** Korean-Color Word Stroop Test

At baseline, no significant differences were observed in the ADAS-cog scores between the αGPC and placebo groups (*p* = 0.329). After 12 weeks, the ADAS-cog scores of both groups significantly decreased from baseline (αGPC: -2.34 ± 3.26, *p* < 0.0001; placebo: -0.97 ± 2.32, *p* = 0.024). The decrease in the ADAS-cog score was significantly greater in the αGPC group (*p* < 0.048) than in the placebo group. This difference remained statistically significant even when the ADAS-non-cog concentration score was added to the analysis of ADAS-cog score (*p* = 0.037). The results are summarized in Table 2 and Fig. 2. Analysis of the memory domain of the ADAS-cog showed that the difference remained statistically significant (*p* = 0.034).

### Secondary outcomes

#### MoCA-K

The MoCA-K includes 7 subscales: visuospatial/executive function, language naming, language-sentence repetition, delayed recall (memory index score), attention, abstraction, and orientation.

At baseline, no significant difference was observed in the total score of MoCA-K and its subscales between the αGPC and placebo groups. In the αGPC group, there was a significant improvement in the total MoCA-K score from baseline, but the difference in improvement between the two groups was not statistically significant (*p* = 0.105). Among the subscales, there was a significantly greater improvement in language-naming scores in the αGPC group than in the placebo group (*p* = 0.003). Both groups showed improvements in memory (delayed recall) score after 12 weeks; however, there was no significant difference in the degree of improvement between the two groups (*p* = 0.844). The results are summarized in Table 2 and Fig. 2.

#### Visual C.P.T

Visual C.P.T includes correct response, omission error, commission error, reaction time, and standard deviation of reaction time.

At baseline, there was no significant difference in any of the measured subscales in visual C.P.T between the αGPC group and placebo group. After 12 weeks, there was a significant decrease in commission error in the αGPC group and standard deviation of reaction time in both groups. However, there was no significant difference in the degree of improvement in any of the items between the two groups. The results are summarized in Supplementary Table 1.

#### K-CWST

In the K-CWST, word reading, color naming, word reading of color words (congruent condition), word reading of color words (incongruent condition), and color naming of color words were analyzed considering duration (time) and error frequency.

At baseline, there was no significant difference between the αGPC and placebo groups on any of the measured subscales of the K-CWST. After 12 weeks, there was a significant decrease in word-reading duration (time) in both groups. Regarding the duration (time) of color naming, there was a significant difference in the decrease between the two groups (*p* = 0.048). The results are summarized in Table 2.

#### S-IADL

S-IADL included potential capacity and current performance.

At baseline, no significant differences were observed in both potential capacity and current performance between the αGPC group and placebo group. After 12 weeks, there was a significant decrease in potential capacity in both groups; however, no significant difference was observed in the degree of decrease between the two groups (*p* = 0.467). The results are summarized in Supplementary Table 1.

#### SCD-Q

At baseline, no significant difference in SCD-Q was observed between the αGPC and placebo groups. After 12 weeks, there was a significant decrease in both groups; however, no significant difference was observed in the degree of decrease between the two groups (*p* = 0.343). The results are summarized in Supplementary Table 1.

#### SGDS-K

At baseline, no significant difference in SGDS-K was observed between the αGPC and placebo groups. After 12 weeks, there was a significant decrease in both groups; however, there was no significant difference in the degree of decrease between the two groups (*p* = 0.560). The results are summarized in Supplementary Table 1.

### Adverse events and safety

No serious AEs were reported, and no participants discontinued the intervention because of AEs. The most common AEs in both the αGPC and placebo groups were gastrointestinal problems, such as abdominal pain, constipation, dyspepsia, and nausea. All AEs reported in both groups resolved completely. Lists of AEs are presented in Supplementary Table 2.

In terms of blood chemistry, uric acid showed a significant increase when compared to the placebo group (*p* = 0.024). Total cholesterol also exhibited a rising trend compared to the placebo group (*p* = 0.033). However, there were no clinically meaningful changes observed in the test group. Results of biological tests are presented in Supplementary Table 3.

## Discussion

This is the first study on αGPC to explore the safety, tolerability, and efficacy of αGPC in overall healthy individuals with concern of MCI. The results show that αGPC is safe and effective in improving cognitive functions in MCI. The decrease in ADAS-cog score was significantly greater at the 12 weeks post-treatment follow-up assessment in the αGPC group than in the placebo group. SHCog™ (600 mg αGPC) once daily was well tolerated by the test group. No serious AEs occurred, and the most frequent side effects included gastrointestinal problems, such as abdominal pain, constipation, dyspepsia, and nausea. However, no significant differences in symptoms were observed between the two groups.

The primary outcome, the ADAS-cog score, showed an average decrease of 2.34 points from baseline after 12 weeks of oral αGPC administration, which was significantly greater than that in the placebo group. The 2.34-point decrease at 12 weeks in our study indicated a slightly greater cognitive improvement than that observed at 12 weeks (decrease of 1.5–2.0 ADAS-cog score) in a RCT investigating the effects of 48 weeks of donepezil treatment [9]. In a 24-week RCT study investigating the effects of regular exercise on study subjects with MCI, the ADAS-cog score decreased by an average of 1.2 points at the endpoint [26]. While the shorter duration of our study makes it difficult to make direct comparisons with these studies, the improvement in ADAS-cog observed in our study suggests comparable effectiveness.

Analysis of the memory domain of the ADAS-cog revealed a significant improvement, compared with that of the placebo group. Previous RCTs investigating the effects of choline supplementation (Citicoline) in older adults with memory problems demonstrated significant enhancements in memory function [27]. Another RCT investigating multi-nutrition, including choline, in prodromal AD also revealed significant improvements in the memory domain [16]. A review of the effects of αGPC on individuals with cognitive decline highlighted the efficacy of αGPC in memory and attention domains [12]. The observed enhancement of memory function by αGPC in our study aligns with these previous findings. Considering the significant relevance of memory decline, particularly in the progression from MCI to AD [6], the memory-enhancing effect of αGPC has crucial clinical significance.

Among the secondary outcome measures in our study, significant improvements in the language-naming subscale of the MoCA-K and the duration (time) of color naming in the K-SVLT were observed in the αGPC group, compared to that in the placebo group. This suggests that αGPC has a particular impact on the language and attention domains. A prospective study evaluating the effects of αGPC on speech recognition in patients with age-related hearing loss indicated its clinical efficacy in central language comprehension, beyond peripheral audibility [28]. A review of the effects of αGPC on patients with dementia highlighted the effect of αGPC on the attention domain [12]. The outcomes of our study provide supporting evidence for existing studies on language and attention functions.

No serious adverse events or drug discontinuation due to AEs were reported in our study. In contrast, an RCT assessing the effects of donepezil on patients with MCI reported drug discontinuation by 42% (165 out of 391) of patients in the donepezil group, with nearly half of them (72 individuals) discontinuing treatment because of AEs [9]. Therefore, the tolerability of αGPC observed in our study might be relatively favorable. Our study results provide additional evidence supporting previous research findings that αGPC possesses a favorable safety profile [29]. After 12 weeks of αGPC intake, there was an increase in uric acid and cholesterol levels; however, no subject showed clinically significant changes [30]. Nevertheless, recent cohort studies have reported an association with stroke [31], long-term monitoring will be necessary for future research.

Our study has several limitations. First, the 12-week duration was not enough to capture the full spectrum of αGPC's potential effects on MCI. Nonetheless, the significant decrease in ADAS-cog scores and the minimal side effects indicate the necessity for longer-term studies on the molecule. Second, the diagnosis of MCI relied solely on clinical assessment tools. Considering that MCI patients with high ADAS-cog scores are at increased risk of progressing to AD [32], additional studies targeting Amyloid-positive (Amyloid +) MCI patients are needed. Additionally, further research using various MCI assessment tools, including those for neuropsychiatric symptoms such as the Mild Behavioral Impairment Checklist [33] or the Neuropsychiatric Inventory-12 [34], is also required. Despite these limitations, our findings suggest the efficacy and safety of αGPC for individuals with aMCI. αGPC is a potential cost-effective therapeutic option for MCI.

The results of our study demonstrate the efficacy and safety of αGPC for MCI. Over a 12-week period, the intervention with αGPC led to a significant improvement in cognitive function. Furthermore, there were no noticeable differences in side effects or tolerability when compared to the placebo group. These findings highlight that αGPC can be considered a management option for whose benefits outweigh its risks in individuals with aMCI.

## Supplementary Information

Additional file 1: Supplementary Table 1. Additional results of neuropsychological tests before and after 12 weeks of choline alphoscerate (αGPC) and placebo administration Reporting guideline for organizational case studies.

Additional file 2: Supplementary Table 2. All adverse event, sorted by affected body system (Safety Set).

Additional file 3: Supplementary Table 3. Biological test results before and after 12 weeks of choline alphoscerate (αGPC) and placebo administration (Safety Set).

## Data Availability

The data used and analysed in this study are available from the corresponding author on reasonable request.

## Abbreviations

αGPC: Alpha glyceryl phosphorylcholine
MCI: Mild cognitive impairment
aMCI: Amnestic mild cognitive impairment
AD: Alzheimer’s disease
ADAS: Alzheimer’s Disease Assessment Scale
ADAS-cog: Alzheimer’s Disease Assessment Scale–cognitive subscale
ADAS-noncog: Alzheimer’s Disease Assessment Scale–non-cognitive subscale
AEs: Adverse effects
RCT: Randomized control trial
SVLT: Seoul Verbal Learning Test
MoCA-K: Korean version of the Montreal Cognitive Assessment
Visual C.P.T: Visual Continuous Performance Test
K-CWST: Korean-Color Word Stroop Test
S-IADL: Seoul-Instrumental Activities of Daily Living
SCD-Q: Subjective Cognitive Decline Questionnaire
SGDS-K: Korean version of the Short Form Geriatric Depression Scale
ITT: Intention-to-treat
