# Supplementary material for: Efficacy and safety of choline alphoscerate for amnestic mild cognitive impairment: a randomized double-blind placebo-controlled trial
Source: BMC Geriatr. 2024 Sep 19;24:774. doi: 10.1186/s12877-024-05366-7 (PMC11412009; doi:10.1186/s12877-024-05366-7)
Supplement: Supplementary file 1 — Additional file 1: Supplementary Table 1. Additional results of neuropsychological tests before and after 12 weeks of choline alphoscerate (αGPC) and placebo administration Reporting guideline for organizational case studies. [file 12877_2024_5366_MOESM1_ESM.pdf]

Supplementary Table 1. Additional results of neuropsychiatric tests before and after 12 weeks of choline alphoscerate (αGPC) and placebo administration

	**αGPC group (N=42)**			**Placebo group (N=32)**			P-value
	Mean (SD)			Mean (SD)			
	Baseline	Week 12	Change	Baseline	Week 12	Change	
**Visual C.P.T**							
Correct response (n)	130.05 (10.13)	132.10 (11.75)	2.05 (8.92)	132.28 (4.42)	133.25 (2.68)	0.97 (3.29)	0.614§
Omission error (n)	4.95 (10.13)	2.90 (11.75)	-2.05 (8.92)	2.72 (4.42)	1.75 (2.68)	-0.97 (3.29)	0.614§
Commission error (n)	5.45 (6.03)	4.19 (4.86)	**-1.26 (3.77)**	5.56 (6.79)	4.28 (5.07)	-1.28 (3.59)	0.960§
Reaction time (s)	0.50 (0.06)	0.50 (0.07)	0.00 (0.04)	0.50 (0.06)	0.50 (0.05)	0.00 (0.03)	0.459§
Standard deviation	0.07 (0.03)	0.06 (0.02)	**-0.01 (0.02)**	0.07 (0.02)	0.06 (0.02)	**-0.01 (0.02)**	0.859§
**S-IADL**							
Current	4.98 (5.15)	2.45 (2.80)	**-2.52 (5.57)**	4.50 (4.65)	2.66 (2.95)	**-1.84 (4.85)**	0.418§
Potential	1.60 (1.62)	0.67 (0.90)	**-0.93 (1.58)**	1.28 (1.42)	0.81 (1.12)	**-0.47 (1.24)**	0.467§
**SCD-Q**	11.64 (6.13)	8.43 (5.70)	**-3.21 (4.14)**	11.41 (5.79)	7.22 (5.10)	**-4.19 (4.60)**	0.343‡
**SGDS-K**	4.62 (3.64)	3.26 (2.91)	**-1.36 (3.36)**	4.09 (3.91)	2.97 (3.33)	**-1.13 (2.81)**	0.560§

Changes between the baseline and week 12 scores were evaluated within each group using the paired t-test. Change values are highlighted in bold for outcomes that were statistically significant ($p<0.05$). Differences in change values between the αGPC and placebo groups were assessed using either the Wilcoxon rank-sum test or the two-sample t-test, and the respective p-values are provided. Statistically significant p-values are shown in bold. ‡: Two sample t-test; §: Wilcoxon rank sum test

αGPC Choline alphoscerate *Visual C.P.T,* Visual Continuous Performance Test; *S-IADL* Seoul-Instrumental Activities of Daily Living; *SCD-Q* Subjective Cognitive Decline Questionnaire; *SGDS-K,* Korean version of the Short Form Geriatric Depression Scale
