# Supplementary material for: Efficacy and safety of choline alphoscerate for amnestic mild cognitive impairment: a randomized double-blind placebo-controlled trial
Source: BMC Geriatr. 2024 Sep 19;24:774. doi: 10.1186/s12877-024-05366-7 (PMC11412009; doi:10.1186/s12877-024-05366-7)
Supplement: Supplementary file 2 — Additional file 2: Supplementary Table 2. All adverse event, sorted by affected body system (Safety Set). [file 12877_2024_5366_MOESM2_ESM.pdf]

Supplementary Table 2. All adverse event, sorted by affected body system (Safety Set)

Body system	αGPC Group (N=52)	Placebo Group (N=48)
	N (%)	N (%)
**Central & peripheral nervous system**		
Dizziness	0	1 (2)
Headache	1 (2)	0
Somnolence	0	1 (2)
**Skeletomuscular**		
Back pain	1 (2)	0
**Gastro-intestinal system**		
Abdominal pain	1 (2)	0
Constipation	0	1 (2)
Dyspepsia	4 (8)	4 (8)
Nausea	0	2 (4)
**Reproductive, female**		
Intermenstrual bleeding	0	0
**Respiratory system**		
Pharyngitis	1 (2)	0
**Skin and appendages**		
Sweating	1 (2)	0
Urticaria	1 (2)	0
**Urinary system**		
Micturition Frequency	0	1 (2)
Pyuria	0	1 (2)
**Miscellaneous**	3 (6)	2 (4)

αGPC Choline alphoscerate
