# Supplementary material for: Efficacy and safety of choline alphoscerate for amnestic mild cognitive impairment: a randomized double-blind placebo-controlled trial
Source: BMC Geriatr. 2024 Sep 19;24:774. doi: 10.1186/s12877-024-05366-7 (PMC11412009; doi:10.1186/s12877-024-05366-7)
Supplement: Supplementary file 3 — Additional file 3: Supplementary Table 3. Biological test results before and after 12 weeks of choline alphoscerate (αGPC) and placebo administration (Safety Set). [file 12877_2024_5366_MOESM3_ESM.pdf]

Supplementary Table 3. Biological test results before and after 12 weeks of choline alphoscerate (αGPC) and placebo administration (Safety Set)

	**αGPC group**			**Placebo group**			*P*-value
	Mean (SD)			Mean (SD)			
	Baseline(N=52)	Week 12(N=50)	Change	Baseline(N=48)	Week(N=42)	Change	
**Hematological Tests**							
WBC (10^3 /μL)	6.08 (1.64)	5.96 (1.43)	-0.05 (1.1)	5.90 (1.39)	4.93 (1.28)	-0.06 (1.07)	0.929‡
RBC (10^6 /μL)	4.34 (0.35)	4.36 (0.36)	0.01 (0.2)	4.40 (1.39)	4.42 (0.39)	0.06 (0.25)	0.242§
Hb (g/dL)	13.28 (0.93)	13.38 (1.08)	0.09 (0.62)	13.49 (1.14)	13.55 (1.09)	0.20 (0.72)	0.680§
Hct (%)	39.77 (2.64)	39.88 (2.84)	0.07 (1.90)	40.30 (3.06)	40.61 (3.17)	0.65 (2.27)	0.180§
Platelet (10^3 /μL)	233.08 (48.64)	234.82 (48.75)	3.24 (22.99)	235.83 (67.56)	231.81 (55.55)	-3.95 (34.29)	0.410§
Neutrophils (%)	56.75 (8.13)	54.01 (8.14)	-2.70 (5.59)	53.70 (7.38)	55.02 (9.10)	0.79 (8.27)	0.093§
Lymphocytes (%)	33.49 (7.43)	35.73 (7.73)	2.24 (4.65)	36.22 (7.09)	34.71 (9.15)	-1.20 (7.59)	0.058§
Monocytes (%)	7.02 (1.78)	7.39 (2.31)	0.36 (2.05)	7.49 (1.55)	8.10 (2.40)	0.61 (2.51)	0.766§
Eosinophils (%)	2.09 (1.52)	2.23 (1.57)	0.12 (1.34)	1.94 (1.54)	1.56 (1.00)	-0.17 (0.94)	0.388§
Basophils (%)	0.66 (0.30)	0.64 (0.31)	-0.02 (0.22)	0.65 (0.30)	0.61 (0.24)	-0.04 (0.27)	0.758§
**Blood Chemistry Tests**							
Glucose (mg/dL)	106.23 (21.53)	111.86 (38.07)	5.36 (27.98)	102.81 (19.32)	102.12 (15.20)	0.17 (11.39)	0.646§
Uric acid (mg/dL)	4.43 (1.03)	4.63 (1.08)	0.21 (0.52)	4.90 (1.53)	4.77 (1.38)	-0.10 (0.72)	**0.024**‡
Total protein (g/dL)	7.17 (0.41)	7.12 (0.34)	-0.03 (0.29)	7.09 (0.33)	7.10 (0.30)	0.03 (0.35)	0.085‡
Albumin (mg/dL)	4.45 (0.21)	4.41 (0.22)	-0.04 (0.20)	4.42 (0.19)	4.41 (0.25)	-0.03 (0.27)	0.343§
Total Bilirubin (mg/dL)	0.76 (0.27)	0.76 (0.32)	-0.01 (0.19)	0.76 (0.33)	0.71 (0.33)	-0.04 (0.18)	0.428‡
AST (IU/L)	22.65 (5.92)	22.24 (4.78)	-0.42 (4.81)	22.63 (7.51)	24.40 (13.93)	24.40 (13.93)	0.431§
ALT (IU/L)	19.31 (8.53)	18.82 (6.99)	-0.38 (6.42)	19.29 (8.75)	21.10 (9.85)	1.31 (8.97)	0.285§
ALP (IU/L)	74.65 (19.99)	75.50 (21.01)	0.86 (7.91)	67.44 (16.89)	69.96 (16.57)	2.31 (8.99)	0.413‡
BUN (mg/dL)	15.70 (3.52)	16.44 (4.62)	0.80 (4.63)	16.65 (4.40)	16.01 (4.10)	-0.62 (3.11)	0.083‡
Creatinine (mg/dL)	0.72 (0.15)	0.75 (0.16)	0.02 (0.07)	0.75 (0.19)	0.76 (0.20)	0.01 (0.06)	0.632§
Total Cholesterol (mg/dL)	173.69 (31.0)	178.64 (36.18)	5.0 (18.32)	186.79 (39.47)	179.26 (38.82)	-5.26 (24.90)	**0.033**§
**Vital Signs, Body Weight**							
Blood pressure (systolic)	136.9 (13.8)	135.6 (13.8)	-0.86 (12.5)	139.23 (13.8)	131.9 (15.4)	-6.40 (14.38)	**0.040**§
Blood pressure (diastolic)	72.71 (8.85)	73.94 (9.50)	1.36 (8.88)	75.02 (9.57)	70.10 (11.55)	-4.02 (10.38)	**0.009**‡
Pulse rate (bpm)	75.58 (12.02)	75.64 (11.90)	0.62 (9.23)	74.79 (10.91)	77.17 (9.84)	2.79 (7.06)	0.216‡
Body weight(kg)	59.04 (10.82)	58.20 (10.57)	-0.67 (1.86)	59.28 (8.12)	58.19 (7.11)	-0.39 (1.63)	0.733§

Differences in change values between the αGPC and placebo groups were assessed using either the Wilcoxon rank-sum test or the two-sample t-test, and the respective p-values are provided. Statistically significant p-values are shown in bold. ‡: Two sample t-test; §: Wilcoxon rank sum test

αGPC Choline alphoscerate *WBC* White Blood Cell; *RBC* Red Blood Cell; *Hb* Hemoglobin; *Hct* Hematocrit; *AST* Aspartate Aminotransferase; *ALT* Alanine Aminotransferase; *ALP* Alkaline Phosphatase; *BUN* Blood Urea Nitrogen; αGPC
